# Current strategies for controlling gastrointestinal nematodes of pigs in Europe

**DOI:** 10.1186/s13071-026-07715-4

**Published:** 2026-10-14

**Authors:** Luise Hohensee, Larissa Oser, Alexandra Laubschat, Josephine Schlosser-Brandenburg, Friederike Ebner

**Affiliations:** 1 Chair of Infection Pathogenesis, Department of Molecular Biology, TUM School of Life Sciences, Technical University of Munich, Freising, Germany; 2 Department of Veterinary Medicine, Centre for Infection Medicine, Institute of Immunology, Freie Universität Berlin, Berlin, Germany; 3 MF 3 Experimental Animal Research and 3R Method Development and Research Infrastructure, Robert Koch Institute, Berlin, Germany; 4 Present Address: Institute of Novel and Emerging Infectious Diseases, Friedrich-Loeffler-Institut, Federal Research Institute for Animal Health, Südufer 10, Greifswald, Insel Riems Germany

**Keywords:** Pig, Gastrointestinal nematode, *Ascaris suum*, *Oesophagostomum* spp., *Trichuris suis*, Helminth control, Pig production

## Abstract

Gastrointestinal nematodes (GIN) remain an often-underestimated challenge in European pig production, affecting animal health, performance, and economic outcomes. The predominant species, *Ascaris (A.) suum*, *Oesophagostomum (O.)* spp., and *Trichuris (T.) suis*, impair growth, feed efficiency, and carcass quality, whilst also contributing to reproductive losses and liver condemnation. Moreover, GIN can modulate host immunity, interfere with vaccine responses, and influence coinfection outcomes, thereby compromising overall herd health. The zoonotic potential of *A. suum* and *T. suis* further highlights the need for effective control strategies. This review critically synthesises the available evidence on the occurrence, impact, and control of porcine GIN in Europe and identifies pig-specific knowledge gaps and priorities for integrated control. GIN occur across all European pig production systems. However, accurate surveillance remains challenging due to methodological heterogeneity, uneven geographical coverage, and limited recent large-scale epidemiological data. Reported farm-level prevalences therefore vary widely, ranging from 1 to 100% (*A. suum*), 2–27% (*O.* spp.), and 0.3–75% (*T. suis*). Higher infection levels are generally reported in outdoor, organic, and small-scale production systems. Current control strategies largely depend on anthelmintics. Reports of reduced efficacy and resistance, particularly in *O.* spp., highlight the need for improved surveillance and diagnostic-based treatment. Hygiene practices, housing design, flooring, bedding, pasture, and herd management can influence transmission, but their effectiveness under commercial conditions remains insufficiently evaluated. Alternative approaches, including dietary interventions, probiotics, genetic selection, and vaccine development, may complement conventional measures, although most are not yet established for routine use. Changes in climate, animal welfare requirements, production systems, and expectations concerning pharmaceutical use may further alter GIN exposure and control needs. Standardised surveillance, contemporary assessments of animal health and economic consequences, field-based intervention studies, and sustainable, integrated control programmes are therefore key priorities for European pig production.

**Graphical Abstract:**

**Figure:**
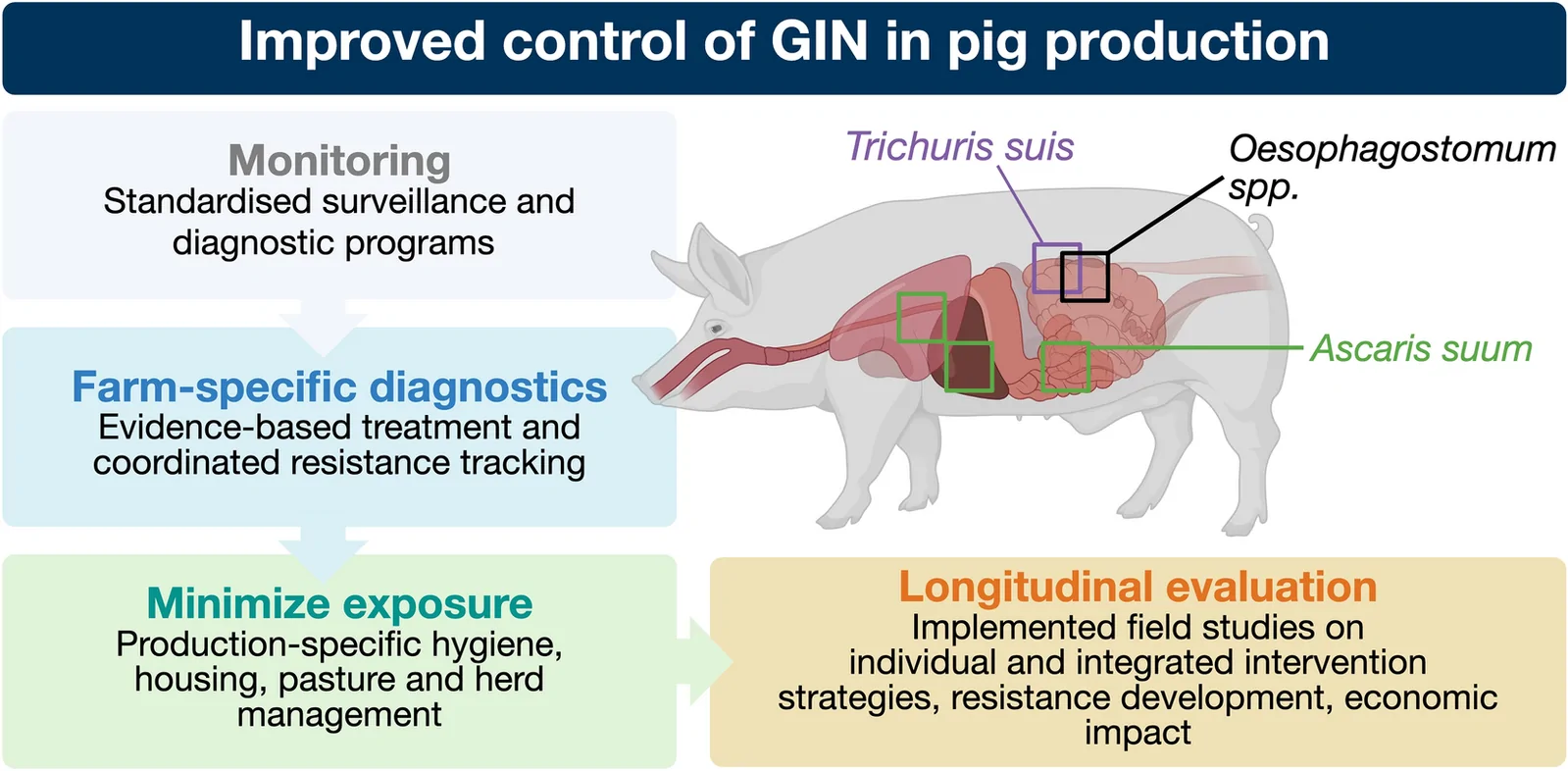

## Background

### Economic impact of gastrointestinal nematode infections

**Fig. 1:**
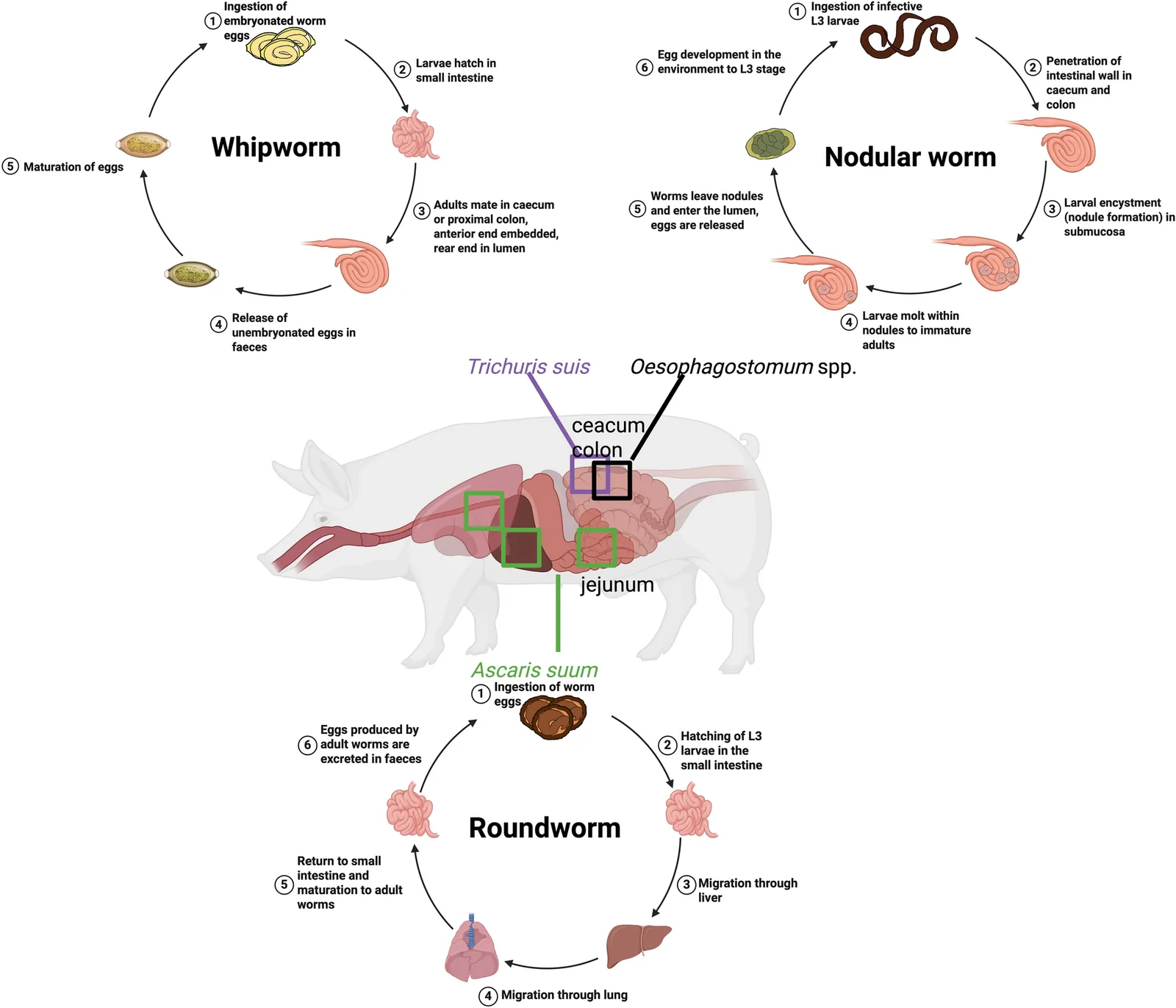
Life cycles and intestinal localization of *Trichuris suis* (whipworm), *Oesophagostomum* spp. (nodular worm), and *Ascaris suum (roundworm)* in pigs. Schematic overview showing the developmental cycles of three major porcine nematodes. *T. suis* develops in the large intestine, where larvae embed in the mucosa of the caecum and colon. *O.* spp. penetrate the caecal and colonic wall to form submucosal nodules before emerging as adults in the lumen. *A. suum* undergoes a hepatotracheal migration before adults mature in the jejunum. Coloured boxes in the pig highlight the anatomical regions affected by each parasite. Created in BioRender

Gastrointestinal nematode (GIN) infections in pigs present a significant challenge to pig production worldwide. In the temperate climate zones of central and northern Europe, *Ascaris suum*,* Oesophagostomum* spp., and *Trichuris suis* are the most commonly observed GIN in pigs [1]. The biological features and life cycle traits of these key nematodes are summarised in Fig. 1 and Table 1.

**Table 1:** Key characteristics of the three most common GINs in European swine herds

	*Ascaris suum*	*Trichuris suis*	*Oesophagostomum* spp.
Highest prevalence in	Fatteners > sows [34]	Young animals (6–38 weeks)[35]	Sows [35]
Eggs	Thick shell with protein layer [35]	Lemon shaped [35]	Thin shell [35]
Development until infectious	4–8 weeks at room temperature [35]	2–3 months in Central Europe (> 1 year possible) [35]	1–3 weeks in warm, moist environments [36]
Tenacity	High (6 years in soil, not influenced by ensiling, embryonation between 5–37 °C) [35, 37, 38]	High (5–11 years in soil; need moisture) [39, 40]	Low (> 10 °C and moisture needed) [35, 41]
Pre-patency	6–8 weeks [35]	6–7 weeks [35]	3–6 weeks (depending on subspecies) [35, 42]

Although these infections are often subclinical, their control is essential to prevent significant impacts on animal health, performance, and economic outcome [2]. Moreover, GIN infections negatively influence key productivity measures, such as average daily weight gain (ADG) and feed conversion rates (FCR) [3–14]. Depending on parasite species and infection intensity, reductions in ADG range from 1 to 31% equating to a daily loss of 60–128 g weight gain and a 4% increase in the time required to reach target weights [4]. Similarly, FCR increases by 14.9–33%, highlighting the inefficiencies GIN infections introduce to production systems [5, 7, 10]. Anthelmintic treatment counteracts these losses, improving both FCR and ADG by 15.1–34.7 g [15–18]. However, some studies have reported inconsistent effects on performance parameters, suggesting that the severity of infection, management practices, and genetic factors may influence the outcome [15–18]. In addition, GIN infections can impair carcass quality*.* Infected pigs show reduced carcass weight (85% of non-excreters), backfat thickness and depth (5.5–12.5%), and meatiness (53.7 in naïve *vs.* 52.1% in GIN-infected pigs) [10, 19–21]. These changes, in turn, directly impact the SEUROP classification, a standardised system used in Europe to evaluate carcass quality based on the percentage of lean meat [22]. Infected carcasses are often downgraded, thus diminishing their market value [10, 19–21]. Additionally, *A. suum* larval migration results in liver condemnation due to granulomas (liver white spots or milk spots), and *O. dendatum* infection in sows has been linked to reduced weaning weight, fewer piglets born, and increased feed consumption during lactation [10, 23]. In contrast, anthelmintic treatment of sows improved reproductive performance, including higher numbers of piglets weaned [24]. Together, these adverse effects of GIN infections impose substantial economic burdens on the porcine industry. Precise estimates are challenging to obtain, but several studies highlight the financial implications arising from GIN infections. Davoudkhani et al. showed that optimising feeding strategies, even without the presence of parasitic infections, yields substantial financial benefits (5% increase in gross margin per pig) [25]. GIN-infested pigs dramatically escalate feeding costs with historical U.S. estimates indicating an additional USD 155 million (approx. EUR 130 million) [6]. Meanwhile, European studies calculated a EUR 60.1 million increase in feeding costs for infected animals in 2010 [26]. Feeding costs further vary between low and high levels of *A. suum* infections. Farms with higher infection rates faced an increase of EUR 14 in feeding costs per pig per day (EUR 0.556 kg/day in low infection intensity *vs.* EUR 0.647 kg/day in high infection intensity). Similar increases in feeding costs have been reported for *T. suis*. Following infection, feeding costs rose from USD 38.76 to USD 39.84–51.43 to reach market weight [7]. *O*. spp. infections only resulted in a mild increase in feeding costs (USD 34.10–36.24 compared to USD 33.86 in uninfected controls) [6]. Moreover, additional expenses arise from the reduction in FCR, decreased lean meat yield, increased susceptibility to respiratory diseases, and the cost of anthelmintic treatment, adding an estimated EUR 5–8 per pig [27, 28]. The price of anthelmintic treatments ranges from USD 0.31 to USD 2.46 (EUR 0.25–2.10) per pig. However, the associated improvements in productivity and carcass quality offset these costs, with benefits estimated to lie around EUR 3 to EUR 12 per finisher [29–31]. Liver condemnation due to *A. suum* infection adds further losses of EUR 0.3–1.13 per kg liver tissue (approximately EUR 2.5 per liver) [15, 32, 33]. Given the high prevalence of *A. suum* and the vast number of pigs slaughtered annually in Europe, total economic losses due to liver condemnation alone were estimated at EUR 17.5 million per year in 2010 [26].

### Animal health implications associated with gastrointestinal nematode infections

Financial losses are a significant driver for addressing GIN infections, but their impact extends far beyond economics. GIN infections are increasingly recognised for their negative effect on vaccine responses and interactions with other pathogens. *A. suum* infections have been associated with increased lung pathology and reduced seroconversion following *Mycoplasma (M.) hyopneumoniae* vaccination [43]. Especially the tissue damage from larval migration through intestinal mucosa, liver, and lungs not only impairs organ function but also increases susceptibility to secondary infections. In fact, *A. suum*-infected pigs demonstrated worsened acute disease after challenge with *Bordetella bronchiseptica* bacterin [44]. Furthermore, *A. suum*-infected pigs showed a reduced pulmonary bacterial clearance after exposure to *Escherichia coli* resulting in more severe pneumonia and systemic disease [45, 46]. Coinfections with *A. suum* have also been linked to increased pulmonary consolidation in conjunction with *M. hyopneumoniae*, as well as elevated mortality and morbidity rates in piglets coinfected with *Actinomyces pyogenes*, and *Pasteurella multocida* [47, 48]. *A. suum* infections have further been associated with higher prevalence and bacterial burden of *Salmonella enterica* spp. in pigs [49, 50]. Viral infections, such as porcine circovirus type 2, are more frequently seen in *A. suum*-infected pigs [51]. Additionally, pigs with hog cholera tend to experience more severe, acute, and longer lasting disease progression [51, 52]. Parasitic infections are often multispecies infections, and several experimental studies have investigated the complex interactions amongst coinfecting parasites. *T. suis* and *O.* spp. coinfections show antagonistic effects as *O.* spp. induce a local immune hypo-responsiveness that promotes its persistence and simultaneously reduces *T. suis* worm counts [53, 54]. In contrast, *O.* spp. and *A. suum* coinfections reduce *O.* spp. worm size and fecundity but increase milk spot formation [55]. Other studies have demonstrated similar antagonistic interactions [56, 57]. Coinfections with *A. suum* and *Trichinella spiralis* resulted in heightened antibody levels and reduced burden of *T. spiralis* muscle larvae, whilst milk spots were exacerbated [58]. In contrast, coinfection of *A. suum* and *Metastrongylus apri* led to moderate disease severity and a reduction in pulmonary worm burdens for both parasites compared with single infections [59]. Prior infection with *Schistosoma japonicum* reduced milk spots in subsequent *A. suum* infections though no effect on adult worm recovery was observed [60].

Notably, much of the experimental evidence on interactions between porcine GIN and bacterial or viral pathogens originates from studies conducted in the 1990s. Although these studies provide important mechanistic evidence that helminth infections can modify susceptibility to, and the clinical outcome of, coinfections, their quantitative relevance under current European production conditions remains uncertain. More recent studies accounting for modern pig genetics, vaccination programmes, housing systems, and pathogen epidemiology are largely lacking.

### Zoonotic risks of gastrointestinal nematode infections

In addition to their direct effects on pig health and productivity, *Ascaris* spp. and *Trichuris* spp. pose a zoonotic risk, emphasising the importance of effective control measures [61, 62]. Numerous cases of human ascariasis have been linked to pig exposure, with individuals living near pigs exhibiting a 4.65-fold higher risk of infection, whilst swine veterinarians were 1.5 times more likely to be seropositive for *A. suum* compared to non-swine veterinarians [63–67]. Interestingly, hybridization between human and porcine *Ascaris* spp. has been reported, and porcine cases of *A. lumbricoides* highlight the bidirectional zoonotic nature of these infections [66, 68–71]. Similarly, *T. suis* infections in pigs have been identified as a potential source of human trichuriasis [72–74]. *Trichuris* spp. were found in Polish sandpits though their exact origin (porcine, canine, or human) remains unclear [75]. Concerns about the spread of GIN in agriculture are growing due to the use of wastewater and sludge as fertiliser [76]. Studies conducted in various European countries have documented the presence of GIN eggs in wastewater and sludge, with concentrations ranging from 0–1 eggs/L in wastewater to <1–867 eggs/g in sludge [77–82]. Furthermore, GIN eggs have been found on fruits and vegetables, raising concerns about the potential for transmission to humans through contaminated produce [83–100]. Additionally, iatrogenic infections have been reported following the therapeutic use of *T. suis* for Crohn’s disease [101]. Although generally asymptomatic, the infection can persist if untreated [101].

In sum, the wide-ranging impact of GIN infections—from economic losses in the pig industry to impaired animal health and potential zoonotic risks—highlights the need for effective and integrated control strategies. Previous strategic reviews have identified broad research priorities for gastrointestinal nematode control across farmed ruminants and pigs, particularly in relation to anthelmintic resistance, diagnostics, vaccines, and the implementation of sustainable control measures [102]. The present review complements this cross-species perspective by focussing specifically on the European pig sector and by integrating evidence on prevalence, production losses, animal health, immune modulation, zoonotic relevance, and currently applicable control measures. Particular attention is given to age, geographical coverage, and comparability of the available evidence, as well as to the implications of recent changes in pig production, animal welfare requirements, and pharmaceutical use. On this basis, we identify pig-specific knowledge gaps and derive priorities for surveillance, research, and integrated GIN control.

### Parasite landscape in European pig production systems

The European Union (EU) currently ranks as the world’s second-largest producer of pig meat, behind China and ahead of the United States [103]. In 2024, the top 5 pig-meat-producing countries in the EU were Spain (5.0 million t), Germany (4.3 million t), France (2.1 million t), Poland (1.9 million t), and the Netherlands (1.4 million t) [104].

Overall, pig production in the EU is characterised by a highly diversified farm structure, a strong export orientation, and ongoing trends towards consolidation. Additionally, higher animal welfare standards and a shift towards organic production further shape the European swine industry [105]. Within the EU, pig production can broadly be categorised into conventional systems, organic production regulated under EU organic legislation, and quality-certified schemes (e.g. Protected Designation of Origin, Protected Geographical Indication) that impose specific requirements on breeds, feeding, and production practices.

Regarding farm sizes, the top 10 EU pig producers (Spain, Germany, France, Denmark, Netherlands, Poland, Italy, Belgium, Romania, and Hungary) show a clear split in average herd size. Intensive integrated farms typically manage large herds, often exceeding 1,000 sows or several thousand finishing pigs as seen in Spain, Germany, and Denmark. As an example, in Germany, roughly 78% of the production pigs live on farms that keep more than 1,000 animals [106, 107]. Conventional indoor farms usually operate on a medium scale, with 100–500 sows per unit. In contrast, organic and extensive systems maintain smaller herds, often below 100 sows, due to space and outdoor access requirements. Smallholder or backyard farms are even smaller, frequently fewer than 10 sows, and are occasionally observed in parts of eastern Europe, such as Romania and Hungary [106].

As part of the European Green Deal, the “Farm to Fork Strategy” articulates several urgent sustainability targets, such as reducing inputs of pesticides and antimicrobials, limiting nutrient surpluses, improving animal welfare, restoring biodiversity, and, crucially, significantly expanding organic farming (European Commission, 2020). These policy directions underpin the growing emphasis on organic and other differentiated production systems alongside conventional schemes. However, despite recent initiatives to promote organic farming, organic pig production remains a niche in the EU, accounting for only about 1% of all pigs in 2023 [108, 109]. At the country level, the highest shares of organic pig production in 2022 are reported from Luxembourg (4.5%), France (4%), Germany (4%), and Austria (3.6%) [104].

Production intensity further distinguishes systems into intensive, characterised by high stocking densities and integrated supply chains (prevalent in Spain, Denmark, and the Netherlands), and extensive, including low-density outdoor systems such as Iberian pig farming in Spain. Housing systems range from fully indoor units with slatted floors to mixed systems with outdoor runs, and permanent outdoor access in organic or traditional systems. Production cycle specialisation adds another layer, with farrow-to-finish farms common in Germany and Poland, whilst Denmark and the Netherlands favour specialised farrowing or fattening units [104]. Additionally, the integration level and biosecurity standards vary amongst EU countries, ranging from highly integrated, high-biosecurity systems in Denmark to smallholder farms with lower biosecurity standards in Romania and Bulgaria. In summary, although conventional systems predominate across the EU, steadily growing organic pig production systems, characterised by outdoor access and organic feed, will necessitate a specific focus on gastrointestinal parasite management as their husbandry conditions inherently increase contact with infective stages and environmental contamination. Understanding how these diverse production systems shape parasite exposure is therefore essential.

### Monitoring strategies and prevalence of gastrointestinal nematodes across Europe

Here, we summarise the available data on the prevalence of the aforementioned three most important gastrointestinal helminths in pigs (*A. suum* (AS), *O. dentatum* (O), and *T. suis* (TS)) in the 10 leading European pork producing countries (Table 2). Hungary, the 10th largest producer, is the only country for which GIN prevalence data in pigs were unavailable.

**Table 2:** Prevalence of *A. suum* (AS), *O. dentatum* (O), *T. suis* (TS), and strongyles (S) in major European pig-producing countries evaluated by *Ascaris* Haemoglobin (As-Hb) ELISA, *Ascaris* L3 larvae ELISA (L3), faecal egg count measured in either McMaster (McM) or FLOTAC chambers, or by milk spot (MS) analysis

Country	Husbandry system/rearing systems	Individual level				Farm level	Method	Year	Sample size	Reference
		Total	Adults	Fatteners	Piglets					
Spain (north)	–	–	–	–	–	MS (*AS*) 25.4% As-Hb (*AS)* 48% L3 (*AS*) 86%	As-Hb L3 MS	2016	21 fattening farms (mean of 2088 pigs/farm)	[129]
Spain (Alicante)	Conventional farming	*AS* 17% *TS* 11% *S* 56%	–	–	–	–	PCR	2006	Pig slurries, 5 farms	[130]
Germany (Bavaria)	Conventional farming	MS (*AS*) 10.8% As-Hb (*AS*) 79%	–	–	–	MS(*AS*) 100% As-Hb(*AS*) 100%	MS As-Hb	2015	15 pigs/farm (serology); 20 farms	[114]
Germany	Alternative pig rearing farms with outdoor areas	*S* 32% *AS* 27% *TS* 5%	*S* 44% *AS* 6% *TS* 1%	*AS* 37% *S* 28% *TS* 9%	*S* 28% *AS* 23% *TS* 1%	*AS* 82% *S* 70% *TS* 35%	FLOTAC (+ nemabiome analysis)	2022–2023	607 faecal samples; 17 farms	[110]
Germany	–	*AS* 6.9%	–	*AS* 6.9%	–	–	MS	2017–2018	24,715 pigs 154 farms	[115]
Germany (North Rhine-Westphalia)	Conventional farming	–	–	–	–	**Pen** *AS* 10.5% *TS* 0.3% *O* 27.5%	McMaster (McM), PCR	1995–1997	13 fattening farms, 229 pen samples	[111]
Germany (North Rhine-Westphalia)	Breeding farm	*O* 55% *AS* 2% *TS* 1%	*O* 55% *A* 2% *TS* 1%	-	-	*O* 79% *AS* 7% *TS* 8%	McM	1993	1,398 sows; 706 farms	[112]
France	Alternative rearing system, farrow-to-finisher/breeder	McM *O* 7.1–35.3% *TS* 2.6–5.1% *AS* 0.5–2.9% As-Hb (*AS)* 43%	**Sow** McM: *O* 35.3% TS 2.6% *AS* 1.8%	McM: *O* 10% TS 4.9% *AS* 2.9%	**Grower** McM: *O* 7.1% TS 5.1% *AS* 0.5%	McM (*O/S*) 47% McM (*AS*) 16% McM *(TS)* 36% As-Hb (*AS*) 91%	McM As-Hb	2020–2021	70 farms (52 farrow-to finish farms; 5 breeders; 13 fatteners)	[113]
Poland	–	*AS* 3.5% *TS* 2.1% *O* 24.2%	**Sows** *AS* 3.8% TS 3.0% *O* 51.8%	**Fatteners** *AS* 6.5% *TS* 1.4% *O* 18.1%	**Suckling piglets** *AS* 0.5% *TS* 0.5% *O* 13.1% **weaners** *AS* 1.2% *TS* 1.2% *O* 12.9%	*AS* 28.6% *TS* 21.4% *O* 68.6% **sows** *AS* 14.3% *TS* 10% *O* 60% **suckling piglets** *AS* 7.1% *TS* 2.9% *O* 35.7% **weaners** *AS* 2.9% *TS* 2.9% *O* 12.9% **fatteners** *AS 15*.*7*% *TS* 4.3% *O* 28.6%	McM	2017	1119 Samples (170 weaners; 215 fatteners, 367 suckling piglets, 367 lactating sows)	[2]
Poland	Close-cycle-pig farm (breeder)	–	**Sows, late gestation** *AS* 21.4% *O* 64.3% **lactating sows** *AS* 35.7% *O* 85.7%	-	*AS* - *O* 7.1%	–	McM	–	14 sows and their offspring, 1 farm; 840 faecal samples	[125]
Netherlands	Free-range (FRF); Organic (OF); Conventional (CF)	–	–	–	–	**FRF** *AS* 8% *O* 4% *TS* 6% **OF** *AS* 8% *O* 3% *TS* 4% **CF** *AS* 1% *O* 2% *TS* 1%	McM	2005	36 farms (FRF 16; OF 11; CF 9)	[127]
Denmark	Organic farms	MS (*AS*) 83–96% McM (*AS*) 12–33%	**Dry sow** McM (*AS*) 13% **lactating** sow McM (*AS*) 12%	**Finisher** McM (*AS)* 33% **starters** McM (*AS*) 19%		MS (*AS)* 100% McM (*AS*) 100%	McM MS	2011	5 farms; 105 livers	[122]
Denmark	Sows in a loose system	–	**Gilts** *AS* 23.1% *O* 7.1% **sows** *AS* 20.8% *O* 8.8%	–	–	*AS* 76% *O* 15%	McM	2009	79 farms, 395 faecal samples	[41]
Denmark	Organic farms	–	**Sows** McM: *O* 20% *AS* 4% *TS* <1%	McM *O* 14% *AS* 33% *TS* 13%	**Weaners** McM: *O* 5% *AS* 28% *TS* 4%	McM *AS* 100% *TS* 75%	McM (MS)	1999	9 farms	[126]
Italy	–	*AS* 7.6% *TS* 1.7%	–	*AS* 7.6% *TS* 1.7%	-	*AS* 45.4% *TS* 27.3%	FLOTAC dual technique	2023	880 pigs 22 farms	[124]
Italy	Feeder-to-finish farrow-to-finish	MS (*AS*) 5.5–98.3% As-Hb (*AS*) 50–100% L3 (*AS)* 54–100%	–	MS (*AS*) 30.4% As-Hb (*AS*) 70.3% L3 (*AS*) 82.2%	–	L3/As-Hb (*AS*) 100%	MS L3 AS-Hb	2022	1,120 pigs (101 blood samples) 9 Farms	[23]
Italy (Tuscany)	–	MS (*AS*) in between batches: 7.6%	–	–	–	151 of 182 batches with MS positive findings	MS	2021	98 farms; 30,961 pigs, 182 batches,	[131]
Italy		MS (*AS)* 26.4%	–	**Heavy pigs:** MS (*AS)* 26.4%	–	92.2%	MS	2020- 2021	754,833 pigs, 399 farms, 5,835 batches	[132]
Belgium	Conventional indoor fattening farms	MS (*AS*) 13.4% As-Hb (*AS*) 61%				MS(*AS*) 95% (19/20) As-Hb(*AS*) 100%	MS; As-Hb	2015	20 pigs/farm (serology); 20 farms	[114]
Austria	Organic farms	MS (*AS*) 50%	–	MS (*AS*) 50%	–	**Finisher unit (faeces):** AS 59% *O* 43% *TS* 27% **Sow unit (faeces):** *O* 66% *A* 30% *TS* 11%	Copromicroscopy (faeces) MS	1999–2000	1,497 fattening pigs for MS; 84 farms for faeces (48 sow units; 51 finishing units)	[123]
Austria	Conventional indoor farm	MS (*AS*) 19.1% As-Hb (*AS)* 68.9% McM (*AS*) 7.4%	–	MS (*AS*) 19.1% As-Hb (*AS*) 68.9% McM (*AS*) 7.4%	–	MS (*AS*) 83.3% As-hb (*AS)* 100%	MS As-Hb mMcM	2019	840 pig livers 18 farms blood/faeces n=177	[28]
Austria	–	MS (AS) 19.9%	–	–	–	–	MS	2021	3,609,619 slaughtered pigs	[121]

Bold labels further detail the study-specific subgroup or unit to which the prevalence values below them refer (e.g.husbandry system, pig category, finisherunit or pen)

Various diagnostic methods were used to determine the prevalence of parasites. The commonly used methods were faecal egg counting methods (FEC), such as (modified) McMaster (McM) and FLOTAC [2, 110–113]. However, in recent years, serological and molecular methods such as ELISA have gained popularity, particularly for detecting *A. suum* [23, 113, 114]. Here, a broader range of tools is available, including *Ascaris* haemoglobin (As-Hb)- and L3-ELISAs, FEC, and necropsy-based assessment of milk spots (MS) [23, 113–115]. The latter stands out as a particularly practical screening tool as it enables large-scale surveillance of pig populations and allows for the detection of acute infection. Meanwhile, the detection of other GIN relies on FECs only [2, 112]. This may underestimate the true prevalence of these parasites as false-negative as well as false-positive egg counting might occur [116, 117].

Based on the data in Table 2, it is difficult to draw a straightforward conclusion about the European prevalence of GIN in pigs due to the high variability in reported prevalence rates. Overall, the farm-level prevalence of *A. suum* ranges from about 1% to 100%. Studies using serological methods have a high seroprevalence at the farm level, ranging from 91 to 100%, whilst individual levels range from 50 to 100% [23, 28, 113, 114], which might indicate a widespread exposure to *A. suum* and a potentially high environmental contamination with infectious eggs. Meanwhile, milk spot prevalence on the individual level varies from 6.9% to 96%, with Germany having the lowest prevalence, at around 6.9% [115] and 10.8% [114]. Recent slaughterhouse numbers from 2024 support these findings, reporting that 3,621,397 livers from fattening pigs were discarded due to the diagnostic finding of hepatitis parasitaria (= milk spots) [118]. Given a total of 43,511,330 slaughtered fattening pigs that underwent the official meat inspection in 2024 [118], this suggests that at least 8.3% of livers were affected by hepatitis parasitaria. Interestingly, data from 2023 indicate a higher prevalence of hepatitis parasitaria findings of approximately 9.8% [118, 119], suggesting a potential decline over time.

Calculations based on the same data source indicate lower prevalence in suckling pigs, with approximately 3.8% in 2024 and 6.1% in 2023, again pointing to a decrease in infection rates. However, the low milk spot prevalence in Germany may reflect meat inspection practices. According to the German meat inspection guidelines in 2007 (AVV LmH 2007), only livers with more than 5 milk spots were classified as having hepatitis parasitaria during meat inspection. Even though this guideline was later revised, this classification was still used as a reference for the meat inspection practice, at least up until 2014 [120]. Although post-mortem inspection of the liver in pigs is regulated (e.g. Article 23 of Regulation (EU) 2019/627), no detailed criteria are defined for the recording or quantification of parasitic liver lesions. Therefore, the recording of hepatitis parasitaria findings at the slaughterhouse may vary between countries, potentially affecting the comparability of prevalence estimates.

Moreover, Austria reported milk spot rates in pigs of 19.1% and 19.9% for 2019 and 2021, respectively [28, 121], and a recent investigation at an Italian slaughterhouse indicated a milk spot prevalence of approximately 30.5% [23]. Overall, Denmark reported the highest milk spot prevalence, from 83 to 96% in 2011 [122]. However, it should be noted that this particular survey was conducted on organic farms. Similarly, an older study from Austria [123] also reported high milk spot rates of around 50% in organic farms. However, surveys that used methods based on FEC generally reported significantly lower prevalences, particularly at the individual level, ranging from approximately 0.5% to 7.6% [2, 28, 112, 113, 124]. Still, a few surveys have observed higher prevalence rates ranging from 12% to 35.7% [110, 122, 125, 126]. Notably, these data were collected either on alternative rearing farms [110, 122, 126] or breeding facilities [125]. At the farm level, prevalence evaluated by FEC methods varies between 1–45.5% [28, 112, 113, 124, 127] and 76–100% [41, 110, 122, 126]. Once again, higher prevalences have been reported in either alternative farming systems or breeding farms, suggesting a heightened risk of infection in these farming systems.

In general, high rates of *O. dentatum* are reported at farm and individual levels, ranging between 27.5–79% and 7.1–85.7%, respectively. An exception at the farm level are two surveys reporting 15% [41] and 2–4% [127]. A comparison of observed *O.* ssp. and other strongyles (S) rates in sows and fattening pigs, which are 20–51.8% and 10%–28%, respectively [2, 110, 126, 128], reveals a higher prevalence in sows. Notably, when considering age, the few studies indicate that younger animals (piglets and weaners) generally exhibit lower prevalence rates, whereas older animals (fatteners and sows) display higher values [2, 113, 125, 126].

In contrast, *T. suis* is consistently reported at lower levels, varying between <1 and 13% on an individual basis although at the farm level, newer prevalence rates usually range between 11 and 36% [2, 110, 113, 123, 124], whereas only a few older surveys reported a lower farm level around 0.3–8% [111, 112, 127] and one study from Denmark a higher prevalence of 75% at the farm level [126].

Over the past decades, data on the prevalence of GIN in swine across Europe, at both the farm and individual levels, have remained limited. Many studies are outdated, methodologically limited, or unrepresentative. Even in Germany, where comparatively more datasets are available, existing studies remain insufficient to provide a comprehensive overview. In addition, many datasets originate from isolated investigations or findings at slaughterhouses rather than from systematic epidemiological studies. Large-scale epidemiological studies of GIN burden across different husbandry systems in swine are almost completely lacking for major pig-producing countries such as Spain. This underlines the urgent need for increased surveillance efforts to gain a better understanding of infection dynamics in the European pig population.

## Control strategies in Europe

### Anthelmintic drugs

According to Regulation (EU) 37/2010, only drugs containing approved active ingredients can be used to treat parasitic infestations in swine in the European Union (Table 3). Hence, all anthelmintic drugs in the EU either contain benzimidazoles, macrocyclic lactones, piperazine or imidazothiazoles. Although various treatment approaches (targeted *vs.* untargeted) exist, routine anthelmintic use appears to negate any influence of application route or treatment strategy on infection prevalence [112, 129]. Treatment of sows pre-farrowing has been described to be especially effective in reducing the prevalence of *O.* spp. and *A. suum* [133].

**Table 3:** Substances authorised for deworming in food-producing species according to Table 1 of Regulation (EU) 37/2010

Active ingredient	Substance class	Field of application (Adults (A) or Larvae (L))	Application	Withdrawal period (meat)
Doramectin	Macrocyclic lactones	*Hyostrongylus rubidus* (A, L), *A. suum* (A, L), *Strongyloides ransomi* (A), *O. dentatum* (A, L), *O. quadrispinulatum* (A, L), *Metastrongylus* spp. (A), *Stephanurus dentatus* (A), ectoparasites	Intramuscular	77 days
Febantel	Benzimidazoles	endoparasites	Oral (feed)	14 days
Fenbendazole	Benzimidazoles	*H. rubidus* (A, L), *O.* spp. (A, L), *A. suum* (A, L), *T. suis*	Oral (feed or water)	4–5 days*
Flubendazole	Benzimidazoles	*A. suum* (A, L), *H. rubidus* (A), *O. dentatum* (A), *T. suis* (A, L), *M. apri* (A), *Strongyloides ransomi* (A, partial effect)	Oral (feed)	14 days
			Oral (water)	4–5 days*
Ivermectin	Avermectins	*A. suum* (A, L4), *H. rubidus* (A, L4), *O.* spp. (A, L4), *S. ransomi* (A, L), *Metastrongylus* spp. (A), ectoparasites, *T. suis* (A, variable effect)	Oral (feed)	12 days
			Subcutaneous	14–35 days*
Levamisole	Imidazothiazoles	*M. apri* (A, L), *Metastrongylus* spp. (L), *A. suum* (A), *S. ransomi* (A), *O.* spp. (A), *H. rubidus* (A, partial effect), *T. suis* (A, partial effect), varying effects on larvae	Oral (feed or water)	14 days
			Intramuscular or subcutaneous	14 days
Oxfendazole	Benzimidazoles	*H. rubidus* (A, L), *O.* spp. (A, L), *A. suum* (A, L), *Metastrongylus* spp. (A, L)	Oral (feed)	14 days
Oxibendazole	Benzimidazoles	*A. suum, Strongyloides* spp., *O.* spp., *T. suis*, *Metastrongylus* spp. (intestinal larvae), *Trichocephalus suis*, *T. spiralis* (A, L), *Macracanthorhynchus hirudinaceus*, *Cysticercus cellulosae*	Oral (feed)	14–28 days*
			Oral (buccal tablet)	28 days
Piperazine		*A. suum* (A), *O. dentatum* (A, variable effect)	Oral (water)	4 days

*withdrawal period dependent on formulation and application route

Overall, prophylactic deworming without prior diagnostics prevails in Europe [134]. This over- or mistreatment of helminths may increase the risk of developing anthelmintic resistance [135, 136]. Resistances have mostly been reported for *O.* spp. Here, reduced efficacy or resistance occurred with both benzimidazoles (flubendazole) and macrocyclic lactones (ivermectin) [137, 138]. No similar effects have been observed for *A. suum* or *T. suis*. It should, however, be noted that deworming efficacy might be dependent on fibre content in the animal feed, with high fibre diets showing stronger efficacy [139].

### Farming infrastructure and management

#### Cleaning and disinfection

Disrupting the faecal–oral transmission of parasite stages is essential for breaking the parasitic life cycle and preventing further infections. This can be achieved by avoiding contact with potentially contaminated faecal matter, which has been reported to reduce the risk of *O.* spp. infection atfarm level [41]. Hence, regular cleaning can effectively reduce the risk of nematode infections as reported for *A. suum* [110] and *O.* spp. [133]. Notably, stables with more uneven, difficult-to-clean surfaces are associated with a higher prevalence of *A. suum* [111]. In structured housing, parasite contamination is distinctly distributed, with latrine areas showing the highest contamination [140]. This should be taken into account during cleaning procedures. A specific focus should be put on appropriate drying periods [141] as low humidity potentially causes eggs to collapse before they become infectious (Wharton et al., 1979 reviewed in [1]). High overall humidity levels may create favourable conditions for gastrointestinal parasites, especially those with thin-shelled eggs like *O.* spp. [1, 41]. Therefore, the type and placement of showers and drinking systems should be scrutinised during risk evaluations.

Disinfection of stables with appropriate antiparasitic disinfectants is also highly relevant to reduce the parasitic burdens within farms. Due to the tenacity of parasitic stages, especially egg stages, a limited number of substances are suitable [142], most of which contain cresols or other highly corrosive ingredients, that can cause severe health problems if ingested or inhaled. Consequently, effective disinfection can only be used in empty barns and is most suitable for all-in all-out management systems.

#### Flooring and bedding

Naturally, flooring and bedding are also important factors in mitigating the risk of helminth infections. Currently, most indoor pigs are either kept on slatted or partially slatted floors, or on solid floors with either shallow or deep litter [143]. In Germany, for example, around 96% of pig housing sites use either fully or partially slatted floors, with fully slatted systems accounting for approximately 79–80% of all housing places. Only about 1% of pig housing provides outdoor access, underscoring the dominance of indoor, slatted-floor systems in conventional German pig production [144]. For the most part, fully slatted, litter-free flooring is associated with a reduced risk of liver condemnation due to *A. suum* milk spots compared to systems using partially slatted floors (less than 50% slatted) or solid floors and bedding [129, 145]. Similarly, *O.* spp. and *Strongyloides* spp. occurrence is reported to be higher on farms with solid, littered floors [2, 133].

Due to growing animal welfare concerns, lameness in particular, fully slatted flooring may be phased out in the future [146, 147]. This could lead to a shift back to partially slatted floors or litter-based free-range systems, which are already widely used in organic farming. In systems with bedding, prevalence is associated with litter depth: Whilst the risk of *T. suis* and Strongyles infection is higher in deep litter [110], *A. suum* and *O.* spp. infestation was found to be higher in shallow than in deep litter [12]. This might be caused by adverse conditions for egg development, such as composting and ammonium content in deep bedding [140, 148].

#### Pasture management

Outdoor access, whether to paddocks or pastures, increases the risks of infection compared to indoor pig production [145]. Whilst parasite species with an indirect life cycle, such as *Hyostrongylus rubidus*, only occur in pasturing herds, outdoor access also increases the risk of infection with *Strongyloides* spp., *O.* spp., *T. suis*, and *A. suum* [2, 110, 112]. Lower pasture stocking rates and strict pasture cleaning might alleviate pasture contamination [122, 149], but so far, no evidence-based recommendations for pasture management exist. Whilst *O.* spp. stages have poor tolerance to freezing, *A. suum* and *T. suis* were reported to persist for at least 4 years [150]. Hence, it has been suggested that long-term (5 to 10 years) pasture rotation is necessary to effectively control contamination [151, 152], which may not be feasible for most farmers. An additional risk factor is the use of swine manure as a fertiliser as dung containing active parasite stages may further contribute to environmental contamination [126, 148, 151]. To reduce that risk, parasitic stages in faeces may be inactivated either by composting [153] or by newer methods, like the use of micro-fungi [154]. Furthermore, it has been hypothesised in the ruminant field that changing the pasture vegetation to specialised crops with anthelmintic properties may be beneficial [155]. However, it remains unclear how that translates to pasturing swine.

### Herd management

Nematode prevalence has been predominantly reported to be higher on smaller farms [2, 112, 124, 133], potentially because professional management practices are more commonly implemented in large-scale farming operations. All-in all-out systems are associated with a lower risk of infection with *Strongyloides* spp., *T. suis*, *O.* spp., and especially *A. suum* [2, 111, 124, 129]; however, they may not be feasible in smaller herds, increasing the risk of GIN infections for smaller farms.

Furthermore, breeding sows are regarded as a prominent source of *A. suum* and *O.* spp. infection for piglets. Hence, frequent replacement of breeding sows and pre-farrowing anthelmintic treatment was shown to reduce prevalence rates, whereas an extended contact with the sow due to late weaning (6 to 9 weeks post-partum) increased prevalence [112, 133, 156]. Overall, finishing units supplied with piglets from multiple farms had a higher risk of nematode infection and milk spot occurrence compared to those raising self-bred piglets [111, 145].

### Alternative approaches

Growing concerns about increasing resistance to anthelmintics and chemical residues within meat products heighten the interest in alternative control approaches, particularly in ecological farming.

Given that all described nematodes inhabit the gastrointestinal tract, feed composition may affect parasite viability and/or fecundity. Indeed, fermentable carbohydrates such as inulin have been shown to reduce worm recoveries and faecal egg counts in pigs infected with *O.* spp. [157] and *T. suis* [158], but not *A. suum*. Substitution of inulin with sugar beet fibres [157] or processed chicory roots [150, 159] reportedly has similar effects. The fermentation of these fibres leads to the production of short-chain fatty acids in the large intestine, which may negatively impact residing parasite stages [150]. Consequently, worms persisting in the small intestine, such as *A. suum*, may be less affected by these feed additives. In line, previous studies have reported variable or only moderate effects [150, 159–161]. Furthermore, the effects of fermentable carbohydrates are not permanent, but reverse quickly after a shift in diet (H. Mejer, 2006 reviewed [150]). Similarly, the use of probiotics during the finishing period has been shown to moderately decrease the risk of milk spot development [145]. A major concern when using plant products is that their composition and the content of active components may be unreliable. Taken together, feed additives are unlikely to serve as a standalone control strategy. However, they may be used to temporarily reduce egg secretion and thereby reduce environmental contamination [1], especially in ecological farming operations, where anthelmintic use is restricted.

Another approach to increase disease resistance against helminth infections in swine herds is selective, marker-based breeding. In sheep and cattle, approaches like mapping economic trait loci (ETL) and using DNA markers for selective breeding have shown promising results [162]. Although similar tools are not yet established for pigs, there is evidence that genetics influence parasite pathogenicity: Hampshire pigs experience greater performance losses from *Strongyloides ransomi* infections than Duroc pigs [163]. Furthermore, studies with Danish–Landrace–Yorkshire–Duroc hybrids revealed strong heritability of resistance to *A. suum* and *T. suis* [164]. In the case of *A. suum* worm burden, total egg excretion levels and antibody levels were genetically influenced, whilst worm size and fecundity were not heritable. For *T. suis*, the highest heritability was observed during the early expulsion phase, suggesting a strong genetic control of the onset of immunity [164]. Therefore, development and implementation of marker-assisted selection could enhance pig resistance to helminth infections, particularly in farming systems where selection for production parameters (e.g. litter size, daily weight gain) is less prioritised.

### Synthesis

The control strategies discussed in the previous sections differ considerably in their evidence base, practical feasibility, and potential for sustained parasite control. No single measure appears sufficient to provide reliable long-term control across the diverse European pig production systems. A comparative appraisal of their potential advantages, limitations, and evidence gaps is provided in Box 1. Building on this assessment, this section critically evaluates the available evidence, identifies the main knowledge gaps, and derives priorities for future GIN control.

**Box 1:** Comparative appraisal of the potential advantages, limitations, and evidence gaps of current and emerging GIN control strategies

Control measure	Advantages	Limitations and evidence gaps
Anthelmintic drugs	o Generally effective when appropriately selected and administered o Fast-acting, particularly against susceptible adult stages o Comparatively easy to integrate into production cycles	o Risk of resistance development o Efficacy varies according to compound, parasite species and developmental stage; no effect on environmental egg stages o Reinfections are common o Risk of inaccurate dosing, particularly during group treatment o Feed composition may affect treatment efficacy
Cleaning and disinfection	o Physical removal of faeces, eggs and free-living larval stages may reduce environmental contamination and disrupt transmission o Does not select for anthelmintic resistance o Repeated and correctly performed procedures may provide sustained reductions in infection pressure	o Labour-intensive and time-consuming o Effect depends on farm infrastructure o Limited availability of agents effective against highly resistant egg stages; many routinely used disinfectants are ineffective o Most effective in all-in/all-out systems; effectiveness is reduced where complete emptying, cleaning and drying of facilities are not feasible o Limited applicability in outdoor/free-range farming o Incorrect use of corrosive disinfectants may pose health risks to users and animals
Flooring and bedding	o Appropriately designed flooring and bedding management may reduce contact with contaminated faeces and facilitate hygiene whilst supporting animal welfare	o Associations between flooring or bedding systems and parasite prevalence are inconsistent o Effects depend on bedding material, depth, moisture and replacement frequency o Welfare-friendly systems may simultaneously increase contact with contaminated organic material
Pasture management	o Lower stocking densities, pasture rotation and sufficiently long resting periods may reduce or dilute infection pressure in extensive systems	o Limited effectiveness against persistent egg stages, particularly those of *A. suum* and *T. suis* o Effectiveness depends on land availability, climate and previous contamination o Difficult to implement in intensive indoor systems o Limited pig-specific evidence defining effective pasture rotation schemes and resting periods
Herd management	o May reduce within-herd transmission through measures such as all-in/all-out management o Sourcing piglets from fewer farms or raising self-bred piglets may reduce the risk of GIN introduction o Diagnostic-based monitoring and treatment of breeding sows may reduce transmission to younger animals	o Requires coordinated planning, staff training and consistent implementation o May require additional space and infrastructure and may not be feasible in all production systems o Comparative field evidence on the effectiveness of individual herd management measures remains limited
Alternative approaches	o May complement conventional measures and reduce reliance on anthelmintics o May provide sustainable or environmentally compatible components of integrated control o Nutritional approaches may complement conventional control measures and temporarily reduce worm burdens or egg excretion o Selective breeding may offer a longer-term option for reducing susceptibility or infection-associated losses	o Heterogeneous group of approaches with widely differing levels of evidence and technological maturity o Most approaches remain experimental and are not standardised for routine use o Efficacy under commercial production conditions is largely unknown o Costs, scalability and logistical implementation remain uncertain

The assessment summarises the evidence of the previous sections. Supporting references are provided in the corresponding sections.

The most fundamental limitation is the lack of recent, representative and methodologically comparable epidemiological data across Europe. The availability and nature of epidemiological data vary between European countries. Germany provides yearly nationwide routine abattoir data on milk spot lesions, offering a valuable indicator of recent *A. suum* larval migration, although these records do not capture all infections and cannot be interpreted as overall GIN prevalence. In contrast, recent studies from Spain and Italy have applied newer, specific serological approaches, including the As-Hb and L3-lung ELISAs, to assess *A. suum* exposure at the farm level [23, 124, 129]. These studies currently provide some of the most detailed information on *A. suum* exposure available in Europe. However, their regional study cohorts limit their national representativeness. At the same time, the interpretation of existing prevalence data is challenging because of the various diagnostic methods used. Studies use copromicroscopy, milk spot assessment, serological, and molecular techniques, with varying sensitivities, specificities, and biological interpretations. Consequently, variations in reported prevalence may be due to methodological differences rather than true epidemiological variation. Thus, the actual distribution and impact of GIN infections within Europe's pig population remain uncertain.

This evidence gap is becoming increasingly relevant because European pig production and thereby the conditions shaping GIN transmission are undergoing substantial structural changes. Apart from the increasing concentration of pig production and changes of the geographical distribution of production within Europe [165], the European pig sector is facing changing market and regulatory conditions: per capita pork consumption in the European Union is expected to stagnate or decline in the future, whilst environmental, animal welfare and biosecurity requirements continue to shape production systems [166].

Changed animal welfare regulations and societal expectations affect housing standards, particularly group housing, stocking density, flooring systems, treatment options (organic husbandry) and access to manipulable materials [167], and may impact parasite transmission and persistence. Additionally, increased use of outdoor runs or pasture-based systems, whilst commendable for animal welfare, may heighten the risk of environmental contamination and parasite persistence [110, 151]. Simultaneously, outdoor access may give rise to other parasite species with indirect lifecycles, such as *Hyostrongylus rubidus* and fundamentally change the parasite landscape. On the other hand, circulating notifiable animal diseases such as African swine fever restrict animal mobility between farms and cause increased biosecurity measures very locally, especially to inhibit contact with wild boars. Together this identifies insufficient understanding of GIN epidemiology in changing production systems with increasing environmental exposure. Furthermore, there is limited knowledge about how these transitions affect parasite transmission, persistence, and control in high-productivity settings.

Emerging anthelmintic resistance poses a threat to sustainable control of GIN infections. Although decreased effectiveness and resistance have been documented, comprehensive data on resistance development or hotspots in and across Europe remain limited due to the lack of systematic monitoring programmes. As a result, the geographical distribution, contributing factors, and molecular mechanisms of resistance continue to be poorly understood.

Furthermore, important uncertainties remain regarding the current animal health and immunological consequences of porcine GIN infections. Evidence on altered vaccine responses, pathogen clearance, and coinfection outcomes is predominantly based on small experimental studies, many of which were conducted several decades ago. The relevance of these findings under current production conditions, therefore, remains unclear. Future experimental studies should account for parasite species, infection intensity and stage, previous exposure, and clinically relevant vaccination or coinfection scenarios. Complementary longitudinal field studies are needed to determine whether experimentally observed immunomodulatory effects translate into measurable differences in vaccine performance, pathogen-associated morbidity, susceptibility to coinfections, or antimicrobial use at the herd level.

Similarly, estimates of the economic consequences of porcine GIN infections remain uncertain and are likely to change with shifts in supply and demand, but many available calculations are based on historical or non-European production systems. Up-to-date opportunity losses and nationwide data are needed to quantify actual production losses and the economic benefits of different control strategies under current European conditions.

Finally, there is a distinct lack of field studies evaluating individual and integrated control strategies. Most epidemiological studies focus on estimating the prevalence of GIN and are valuable in that regard. They, however, provide limited evidence on whether changes in management or implemented control strategies reduce infection at the animal or farm level. Longitudinal, farm-based intervention studies are therefore needed to evaluate the effects of a stepwise implementation of parasite control measures under commercial production conditions. These studies should combine standardised meat inspection findings with parasitological and/or serological sampling as well as detailed risk analysis. Furthermore, the effectiveness of control measures beyond anthelmintic treatment should be assessed to offer guidance on housing design, hygiene practices, pasture management, integrated control strategies, and biosecurity measures in the future.

In sum, the key knowledge gaps highlighted by this review reflect a larger issue: parasite control guidelines are increasingly used in production systems that differ from the conditions under which much of the existing evidence was obtained. Filling these gaps is crucial for creating effective, cost-efficient, and sustainable GIN management strategies for future European pig production systems.

## Outlook: priorities for future GIN control in European pig production

The effective management of gastrointestinal helminths in pigs remains challenging and is further complicated by climate change, globalisation, and shifting societal expectations. Modern demands for sustainable and welfare-oriented farming systems pose additional difficulties. Organic and free-range systems promote animal welfare but often increase the exposure to parasitic nematodes due to greater environmental contact [1, 41, 110, 127, 150, 151, 160, 168]. The preference for meat with reduced reliance on pharmaceutical treatments further pressures producers to adopt natural deworming strategies, paddock rotation, and alternative treatments. Balancing these expectations with effective parasite control and environmental sustainability requires innovative approaches in veterinary medicine and farm management. Together, these developments create interrelated challenges for sustainable GIN control, which are summarised alongside the associated outstanding questions in Box 2.

**Box 2:** Current challenges, priorities for future GIN control, and associated outstanding questions

Current challenge	Priority for future control	Outstanding question
Fragmented and heterogeneous surveillance data	o Establish harmonised farm- and slaughterhouse-based surveillance across major European pig-producing countries	How can standardised surveillance generate current, representative, and comparable prevalence data across Europe?
Limited availability and comparability of diagnostic tools	o Standardise sampling and diagnostic methods and develop practical tools for diagnostic-based treatment	Which diagnostic approaches are sufficiently sensitive, reliable, and feasible for routine use under commercial conditions?
Uncertain distribution of anthelmintic resistance	o Implement coordinated monitoring of anthelmintic efficacy and emerging resistance	How widespread is reduced anthelmintic efficacy in European pig production, and how can emerging resistance be detected at an early stage?
Outdated evidence on animal health and economic consequences	o Generate contemporary experimental and longitudinal field data	To what extent do porcine GIN infections affect vaccine performance, coinfection outcomes, morbidity, antimicrobial use, and production losses under current European conditions?
Changing production and environmental conditions	o Adapt GIN prevention to welfare-oriented housing, outdoor access, and climate-related changes	How do changing housing, management, and climatic conditions alter GIN transmission, and how can resulting infection risks be mitigated?
Persistent environmental contamination and reinfection	o Develop and validate effective combinations of cleaning, disinfection, flooring, bedding, and pasture management	Which environmental interventions sustainably reduce contamination with persistent GIN stages under field conditions?
Limited field evidence for integrated control	o Evaluate integrated, farm-specific programmes combining surveillance, treatment, and management measures	Which combinations of diagnostic-based treatment, hygiene, herd management, and environmental interventions are effective, feasible, and economically sustainable?
Limited availability of complementary control tools	o Advance and validate nutritional, genetic, and vaccine-based approaches	Which complementary approaches provide reproducible benefits under commercial conditions and can reduce reliance on anthelmintics?
Implementation gap between evidence and practice	o Translate evidence into farm-specific guidance and coordinated policy support	How can effective control programmes be implemented through cooperation between farmers, veterinarians, researchers, industry, and policymakers?

To overcome these challenges, we propose that future GIN control should move towards integrated approaches rather than relying primarily on prophylactic treatment. First, regular standardised GIN monitoring should be established, ideally integrating farm-level sampling at slaughterhouses. Defining standardised lesion categories and EU-wide reporting procedures could further improve surveillance and the comparability of *A. suum* monitoring across European countries. Complementary serological surveillance could be implemented alongside meat inspection to identify GIN exposure that may not be reflected by visible liver lesions. These systems could provide a timely, representative overview of parasite prevalence, particularly in countries with major pork production. Furthermore, automated, artificial intelligence-based methods, PCR, proteomics, and biomarker-based approaches may supplement and advance current diagnostic methods [169].

Second, anthelmintic treatment should be guided by diagnostic information rather than prophylactic treatment. Experiences from well-established parasite control systems in horses demonstrate the value of targeted treatment approaches. Additionally, reduced drug efficacy should be monitored, and anthelmintic resistance tracked routinely. Close communication between farmers, veterinarians, academia, and the pharmaceutical industry is crucial to identify emerging resistances at an early stage and respond appropriately.

Third, farming infrastructure and management should be integrated into GIN control. Stables should allow for effective cleaning and disinfection. Easy-to-clean surfaces, adequate drainage, and sufficient drying periods may help reduce contamination within the stables. Where pigs have access to outdoor areas, parasite control should be a key part of managing these spaces. Runs and pastures should be protected against contamination from slurry, manure, and wild animals, including wild boar. Where feasible, pasture rotation and sufficiently long resting periods should be implemented. Furthermore, the use of swine manure as fertiliser in areas accessible to pigs should be avoided. Additionally, future research should determine appropriate pasture treatment strategies to alleviate parasite burden. Where possible, animals should be handled in all-in/all-out systems to limit the transmission of GIN within farms. Additionally, breeding sows should be monitored regularly and, if indicated, treated before entering farrowing groups. Animals introduced from external sources should originate only from monitored herds and should be quarantined and tested before being integrated into the resident population.

Finally, the development of alternative control strategies should be pursued as a long-term strategy. Particularly, the development of vaccines against porcine GIN should remain a research priority although the biological and practical barriers to effective antiparasitic vaccination are substantial. Progress with native and recombinant vaccines against gastrointestinal nematodes of ruminants provides useful conceptual and technological guidance [170, 171], but direct transferability to pigs cannot be assumed. Research priorities include the identification of stage-specific protective antigens, the definition of correlates of protection, the identification of suitable adjuvants and delivery systems, and the assessment of efficacy under repeated low-dose exposure and commercial production conditions. Given the current absence of a market-ready vaccine, vaccination should be considered a future component of integrated control rather than a short-term replacement for diagnostics, management, and targeted anthelmintic treatment.

## Conclusions

In conclusion, the control of GIN in pigs faces significant challenges. Advancements in diagnostics and alternative treatment strategies offer a viable path forward. Collaborative efforts amongst veterinary research, farm management, and societal stakeholders are essential to alleviate parasitic pressure and ultimately improve overall animal health.

## Data Availability

Data supporting the main conclusions of this study are included in the manuscript.
